# Optimizing treatment selection, and sequencing decisions for Management of HR-Positive, HER2-Negative advanced breast cancer – Proceedings from breast cancer expert group meeting

**DOI:** 10.1186/s12919-021-00224-5

**Published:** 2021-08-09

**Authors:** Shaheenah Dawood, Maria Konstantionva, Rebecca Dent, Florencia Perazzo, Sung-Bae Kim, Cynthia Villarreal-Garza, Sandra Franco, Ming-Shen Dai, Sergio Simon

**Affiliations:** 1 Dubai Health Care City, Consultant Medical Oncologist, Mediclinic City Hospital - North Wing, Dubai, UAE; 2 Head of the Department of antitumor drug therapy, F. VladimirskIy Moscow Regional Research Clinical Institute (MONIKI), Moscow, Russia; 3 Head, Breast Medical Oncology Team, National Cancer Center Singapore, Singapore, Singapore; 4 Department of Oncology, Centro de Educación Médicae Investigaciones Clínicas (CEMIC), Ciudad de Buenos Aires, Argentina; 5 Department of Oncology, Asan Medical Center, University of Ulsan College of Medicine, Songpa-gu, Seoul, South Korea; 6 Centro de Cancer de Mama, Hospital Zambrano Hellion, Tecnologico de Monterrey, San Pedro Garza García, NL Mexico; 7 Depto. de Investigacion, Instituto Nacional de Cancerologia, Mexico city, Mexico; 8 Head of Oncology, Clínica del Country, Bogotá, Colombia; 9 Department of Hematology/Oncology, Tri-Service General Hospital, National Defense Medical Center, Taipei City, Taiwan; 10 Centro Paulista de Oncologia (CPO), Sao Paulo, Brazil

**Keywords:** Cyclin-dependent kinase 4/6 inhibitor, Endocrine therapy, Hormone receptor-positive, Human epidermal growth factor receptor 2 negative, Metastatic breast cancer PI3K/mTOR inhibitor, Registry, Sequence of therapy

## Abstract

**Purpose:**

The therapeutic landscape of hormone receptor-positive (HR+), human epidermal growth factor receptor 2-negative (HER2−) metastatic breast cancer (mBC) has evolved considerably with the introduction of newer targeted agents and their combinations with endocrine therapies. In this scenario, optimizing treatment selection and sequencing is daunting for clinicians. The purpose of this review is to provide evidence-based answers to key clinical questions on treatment selection and sequencing for the management of HR + HER2 − mBC.

**Design:**

A panel of nine key opinion leaders from Argentina, Brazil, Colombia, Mexico, Moscow, Singapore, South Korea, Taiwan, and UAE convened in October 2018. They reviewed the literature and formulated answers to clinical questions on optimizing the management of HR + HER2 − mBC.

**Results:**

Evidence-based answers were formulated for: (1) optimal initial treatment choice; (2) ovarian function suppression, optimal endocrine partner, and role of cyclin-dependent kinase 4/6 (CDK4/6) inhibitors (in premenopausal women); (3) better first-line standard of care than aromatase inhibitors; (4) preferred second-line treatment; (5) treatment of oligometastatic disease; (6) factors influencing first-line single-agent endocrine therapy choice; (7) influence of endocrine resistance on treatment selection; (8) optimal maintenance regimen in visceral crisis; and (9) need for a breast cancer registry for patients with HR + HER2 − mBC. The panel also proposed a treatment-sequencing algorithm for the management of HR + HER2 − mBC.

**Conclusion:**

The current article will serve as a comprehensive guide for optimizing the management of HR + HER2 − mBC. The proposed breast cancer registry will help identify unmet needs and develop strategic regional policies to help improve access to optimized care for HR + HER2 − mBC.

## Background

In 2018, breast cancer was the most commonly diagnosed cancer worldwide (24.2%) and in about 154 countries; it was also the leading cause of cancer death globally (15%) and in over 100 countries among women [1]. The high mortality rate associated with breast cancer is attributed to the complications resulting from advanced disease. While about 30% of women diagnosed with early breast cancer eventually progress to develop advanced disease, 6% of cases present with de novo metastatic breast cancer (mBC) at initial presentation [2, 3]. The prognosis of mBC is poorer compared to that of localized breast cancer [3].

The main subtypes of breast cancer based on gene profiling include: (1) luminal cell-like tumors; (2) basal cell-like (BCL) tumors; and (3) human epidermal growth factor receptor 2 (HER2)-overexpressing tumors [4]. Based on immunophenotypic characteristics, breast cancer may be divided into: (1) estrogen receptor (ER) + and/or progesterone receptor (PR)+, and HER2− tumors (luminal A); (2) ER+ and/or PR+, and HER2+ or HER2− tumors (luminal B); (3) ER−, PR−, and HER2− tumors (triple-negative); and (4) ER−, PR−, and HER2+ tumors (HER2-overexpressing) [4]. Luminal A breast cancer is the most common subtype [5, 6].

The therapeutic landscape of advanced luminal A (HR + HER2−) breast cancer has evolved dramatically in recent years, with the introduction of several new targeted treatment regimens. With the availability of newer regimens, clinicians are now faced with the challenge of optimizing treatment selection and sequencing for the first and subsequent lines of treatment for HR+ HER2 − mBC.

### Rationale and objectives of expert group meeting

Given the high incidence of and emerging evidence on newer treatment regimens for HR + HER2 − mBC, an expert panel of nine key opinion leaders from Argentina, Brazil, Colombia, Mexico, Moscow, Singapore, South Korea, Taiwan, and UAE convened in October 2018 in Munich, Germany to: (1) review the current treatment armamentarium; (2) discuss and formulate answers to key clinical questions on optimizing treatment; and (3) examine the feasibility of developing a regional breast cancer registry.

### Optimizing treatment of HR + HER2 − mBC: key clinical questions and potential answers

#### Clinical question 1

What is the optimal choice for the initial treatment of HR + HER2 − mBC—endocrine therapy or chemotherapy?

International mBC guidelines and evidence from Cochrane database analyses recommend endocrine therapy as the preferred first-line option for the treatment of HR + HER2 − mBC, even in the presence of visceral disease—except in patients with proof of visceral crisis, immediate life-threatening disease or endocrine resistance [7–10]. Current endocrine therapy regimens for the management of HR + HER2 − mBC include selective ER modulators (tamoxifen), selective ER downregulators (fulvestrant), aromatase inhibitors (AIs) (steroidal AI: exemestane; nonsteroidal AI [NSAI]: letrozole and anastrozole), and combination of endocrine therapies with targeted therapies such as cyclin-dependent kinase (CDK) 4/6 inhibitors (palbociclib, abemaciclib, and ribociclib), mammalian target of rapamycin (mTOR) inhibitors (everolimus) [11] and phosphoinositide-3-kinase, catalytic, alpha-polypeptide (*PIK3CA*) inhibitors (alpelisib) [12].

Tamoxifen is one of the earliest first-line standards of care for the management of HR + HER2 − mBC [13–15]. It has a better safety profile compared to other conventional HR + mBC treatments available, before the introduction of AIs [16, 17]. However, the emergence of clinical evidence supporting the superior efficacy of AIs over tamoxifen for the treatment of HR + mBC (Table 1) led to a shift in the first-line standard of care from tamoxifen to AIs [18–22, 39].

**Table 1:** Summary of key studies for the first-line treatment of women with HR+, HER2– or unknown HER2 status mBC

First author [year] [study name]	Treatment arms [n]	Key endpoint outcomes
Mouridsen H et al.*,* 2001, 2003 [International Letrozole Breast Cancer Group]^a^ [18, 19]	• Letrozole 2.5 mg OD [*n* = 453] • Tamoxifen 20 mg OD [*n* = 454]	**Results at final 32 mos FU—Letrozole/tamoxifen** TTP = 9.4/6.0 mos; *p* < 0.0001 OS = 34/30 mos; *p =* NS
Bonneterre J et al.*,* 2000 [TARGET]^a^ [20]	• Anastrozole 1 mg OD [*n* = 340] • Tamoxifen 20 mg OD [*n =* 328]	**Anastrozole/tamoxifen** *p =* NS for all TTP = 8.2/8.3 mos
Nabholtz JM et al.*,* 2000 [the North American trial]^a^ [21]	• Anastrozole 1 mg OD [*n =* 171] • Tamoxifen 20 mg OD [*n* = 182]	**Anastrozole/tamoxifen** TTP = 11.1/5.6 mos; *p* = 0.005
Paridaens RJ et al.*,* 2008 [EORTC BCCG]^a^ [22]	• Exemestane 25 mg OD [*n =* 182] • Tamoxifen 20 mg OD [*n* = 189]	**Exemestane/tamoxifen** PFS = 9.9/5.8 mos; *p* = 0.028
Robertson JF et al.*,* 2016 [FALCON] [23]	• Fulvestrant 500 mg IM on days 0, 14, and 28, and every 28 days thereafter [*n* = 230] • Anastrozole 1 mg OD [*n* = 232]	**Fulvestrant/anastrozole** PFS: 16.6/13.8 mos; *p* = 0.05 PFS in patients with nonvisceral disease: 22.3/13.8 mos (hazard ratio, 0.59; 95% CI, 0.42–0.84)
Bergh J et al.*,* 2012 [FACT]^a^ [24]	• Anastrozole 1 mg OD [*n* = 256] • Anastrozole 1 mg OD plus fulvestrant 500 mg IM on day 1 and 250 mg on days 15 and 29 of first cycle, and every fourth week thereafter [*n* = 258]	**Fulvestrant + anastrozole/anastrozole** TTP: 10.8/10.2 mos; *p =* NS OS: 37.8/38.2 mos; *p =* NS
Mehta RS et al.*,* 2012, 2019 [SWOG 0226]^b^ [25, 26]	• Anastrozole 1 mg OD [*n* = 345] • Anastrozole 1 mg OD plus fulvestrant 500 mg IM on day 1 and 250 mg on days 14 and 28 of first cycle, and 28 days thereafter [*n* = 349]	**Anastrozole/fulvestrant + anastrozole** **All patients** PFS:13.5/15 mos; *p =* 0.007 Final OS: 42/49.8 mos; *p =* 0.03 **Patients with no prior tamoxifen** PFS:12.6/17 mos; *p =* 0.006 OS: 40.3/52.2 mos (HR 0.73, 95% CI 0.58–0.92) **Patients with prior tamoxifen** PFS: 14.1/13.5; *p* = 0.37 OS: 43.5/48.2 mos; *p* = 0.09
Finn RS et al.*,* 2016 [PALOMA-2] [27]	• Letrozole 2.5 mg OD [*n* = 222] • Letrozole 2.5 mg OD + palbociclib 125 mg OD for 3 weeks followed by 1 week off [*n* = 444]	**Letrozole + palbociclib/letrozole:** PFS: 24.8/14.5 mos; *p* < 0.001
Hortobagyi GN et al., 2016, 2018; O Shaughnessy J et al.*,* 2018; Sonke GS et al.*,* 2018 [MONALEESA-2] [28–31]	• Letrozole 2.5 mg OD + Placebo [*n* = 334] • Letrozole 2.5 mg OD + ribociclib 600 mg per day on a 3-weeks–on, 1-week–off schedule in 28-day treatment cycles [*n =* 334]	**Letrozole + ribociclib/letrozole + placebo** PFS: 25.3/16.0 mos; *p* < 0.0001 ORR: 42.5%/28.7%; *p* = 0.00009 **De novo mBC patients** PFS: Not reached/16.4 mos; HR, 0.45; 95% CI 0.27–0.75 **Elderly patients (≥65 years)** PFS: Not reached/18.4 mos; HR, 0.608; 95% CI 0.394–0.937
Goetz MP et al., 2015; Goetz MP et al., 2017; Johnston S et al., 2019 [MONARCH-3] [32–34]	• Anastrozole 1 mg or letrozole 2.5 mg OD + placebo [*n* = 165] • Anastrozole 1 mg or letrozole 2.5 mg OD + abemaciclib 150 mg orally every 12 h till progression [*n* = 328]	**Abemaciclib + NSAI/NSAI + placebo** PFS at interim analysis: Not reached/14.7 mos; *p* = 0.000021 Final PFS: 28.1/14.7 mos; *p* = 0.000002 ORR: 61%/45.5%; *p* = 0.003
Slamon DJ et al.*,* 2018 [MONALEESA-3]^c^ [35]	• Ribociclib 600 mg per day on a 3-weeks–on, 1-week–off schedule in 28-day treatment cycles + fulvestrant 500 mg IM on day 1 of each 28-day cycle, with an additional dose on day 15 of cycle 1 [total, *n* = 484; first-line setting, *n* = 238] • Fulvestrant + placebo [total, *n* = 242; first-line setting, *n* = 129]	**Ribociclib + fulvestrant/fulvestrant + placebo:** Overall PFS (first-line settings): 33.6/19.2 mos; HR 0.546, 95%CI 0.415–0.718 OS (first-line settings): Not reached/45.1 mos; HR 0.700, 95% CI 0.479–1.021
Tripathy D et al., 2018; Im SA et al., 2019 [MONALEESA-7]^c^ [36]	• Ribociclib 600 mg per day on a 3-weeks–on, 1-week–off schedule in 28-day treatment cycles + oral tamoxifen (20 mg/day)/NSAI + goserelin [total, *n* = 335; first-line setting, *n* = 208] • Placebo + oral tamoxifen (20 mg/day)/NSAI + goserelin [total, *n* = 337; first-line setting, *n* = 196]	**Overall: Ribociclib + endocrine therapy/placebo + endocrine therapy** OS at 42 months: 70.2%/46.0%; *p* = 0.000973 **Subgroup: Ribociclib + tamoxifen/placebo + tamoxifen** PFS: 22.1/11 mos; hazard ratio 0.59; 95% CI 0.39–0.88 OS at 42 months: 71.2%/54.5%; hazard ratio 0.79; 95% CI 0.45–1.38 **Subgroup: Ribociclib + NSAI/placebo + NSAI** PFS: 27.5/13.8 mos; hazard ratio 0.57; 95% CI 0.44–0.74 OS at 42 months: 69.7%/43%; hazard ratio 0.70; 95% CI 0.50–0.98
Royce M et al., 2018 [BOLERO-4]^c^ [37]	• Everolimus 10 mg/day + letrozole 2.5 mg/day [first-line setting, *n* = 202]	**Everolimus + letrozole:** PFS: 22 mos
Beck JT et al.*,* 2014 [Exploratory analysis of BOLERO-2] [38]	• Everolimus 10 mg OD + exemestane 25 mg OD [*n* = 100] • Exemestane 25 mg OD [*n* = 37]	**Everolimus + exemestane/exemestane:** PFS: 11.5/4.1 mos PFS [according to central assessment]: 15.2/4.2 mos

*HR* Hormone receptor, *HER* Human epidermal growth factor receptor, *mBC* Metastatic breast cancer, *OD* Once-daily, *FU* Follow-up, *mos* Months, *NS* Not significant, *PFS* Progression-free survival, *TTP* Time to treatment progression, *OS* Overall survival, *CI* Confidence interval, *IM* Intramuscular, *ORR* Objective response rate, *NSAI* Nonsteroidal aromatase inhibitor, *TARGET* Tamoxifen or arimidex randomized group efficacy and tolerability study, *EORTC* European Organisation for the Research and Treatment of Cancer, *BCCG* Breast Cancer Cooperative Group, *FALCON* Fulvestrant and AnastrozoLe COmpared in hormonal therapy Naïve advanced breast cancer, *FACT* Fulvestrant and Anastrozole Combination Therapy, *SWOG* SouthWest Oncology Group, *PALOMA* Palbociclib ongoing trials in the management of breast cancer, *MONARCH* The Study of Abemaciclib [LY2835219] Combined With Fulvestrant in Women With Hormone Receptor Positive HER2 Negative Breast Cancer, *MONALEESA* Study of Efficacy and Safety of LEE011 in Postmenopausal Women With Advanced Breast Cancer, *BOLERO* The breast cancer trials of oral everolimus

^a^HER2 status not reported

^b^90.5% HER2– patients

^c^Included both first- and second-line settings

Other currently available endocrine therapy-based regimens for the first-line treatment of HR + HER2 − mBC include fulvestrant; combination therapy of CDK4/6 inhibitors plus AI/fulvestrant/tamoxifen; combination therapy of everolimus plus AI/fulvestrant; and combination therapy of fulvestrant plus NSAI [7–9, 40]. Specifically, (CDK) 4/6 inhibitors plus endocrine therapy are commonly used as a first-line therapy – palbociclib, ribociclib, and abemaciclib have shown to improve progression-free survival (PFS) in combination with endocrine therapy [41]. Further, ribociclib and abemaciclib have also shown overall survival (OS) benefit in combination with endocrine therapy in separate trials [40, 42, 43]. In a meta-analysis of nine RCTs including more than 5000 patients, CDK 4/6 inhibitors in combination with endocrine therapy versus endocrine therapy alone were associated with improved PFS (hazards ratio [HR] 0.54, 95% confidence interval [CI] 0.50–0.59; *p* < 0.00001) and OS (HR, 077 95% CI 0.69–0.85; *p <* 0.00001), but increased risks of neutropenia, leukopenia, and diarrhea [44].

#### Clinical question 2

What are the recommendations for ovarian function suppression or ablation in premenopausal women with HR + HER2 − mBC? What is the best partner hormonal agent in this setting?

According to American and European guidelines, premenopausal women with HR + mBC should be offered ovarian suppression with gonadotropin-releasing hormone (GnRH) agonists or ablation with oophorectomy in combination with endocrine therapy [7, 9]. Endocrine therapy with tamoxifen, ovarian suppression, or ablation alone may also be considered in premenopausal patients with HR + mBC who have not been exposed to prior hormone therapy [7]. However, combination therapy has been found to be significantly superior to GnRH agonist or tamoxifen therapy alone, in terms of progression-free survival (PFS; 9.7 months vs. 6.3 or 5.6 months, respectively, *p* = 0.03) and overall survival (OS; 3.7 years vs. 2.5 years or 2.9 years, respectively, *p* = 0.01) [45].

Other preferred first-line single-agent endocrine therapy options in combination with ovarian suppression or ablation for the treatment of premenopausal women with HR + mBC are AIs and fulvestrant [9]. The use of AIs alone without ovarian suppression or ablation is contraindicated in premenopausal women due to a risk of induction of ovulation [7]. However, the combination of AIs plus ovarian suppression has been found to be effective and safe for the first-line treatment of premenopausal women with HR + mBC [46, 47], with a median TTP and duration of clinical benefit of 12 and 24 months, respectively, in initial clinical studies [46]. The combination of AI plus ovarian suppression has also been found to be safe and effective for premenopausal women with HR + mBC refractory to tamoxifen plus ovarian suppression or in whom tamoxifen is contraindicated [48]. Fulvestrant is another emerging option for the treatment of premenopausal women with HR+ HER2 − mBC, including those who are refractory to tamoxifen [49, 50].

#### Clinical question 3

Are CDK4/6 inhibitors a feasible treatment option in premenopausal women with HR + HER2 − mBC?

Emerging evidence from MONALEESA-7, PALOMA-3, and MONARCH-2 supports the use of CDK 4/6 inhibitors in combination with endocrine therapy and ovarian suppression for the first- and second-line treatment of premenopausal women with HR + HER2 − mBC.

MONALEESA-7 is a phase III, randomized, double-blind, placebo-controlled trial in which 672 premenopausal or perimenopausal women with HR + HER2 − mBC with or without prior endocrine therapy or chemotherapy were randomized to receive a CDK4/6 inhibitor (ribociclib) or placebo along with either tamoxifen or NSAI (letrozole or anastrozole), both groups being treated with goserelin [36, 40]. The median PFS in the ribociclib group was significantly higher than that noted in the endocrine therapy plus ovarian suppression group (23.8 vs. 13 months, respectively; hazard ratio [HR] 0.55, 95% confidence interval [CI] 0.44–0.69; *p* < 0.0001) [40].

PALOMA-3 is another phase III, randomized, double-blind, placebo-controlled trial that included both post- (79.3%) and pre−/perimenopausal (20.7%) HR + HER2 − mBC women who had progression or relapse during previous endocrine therapy. Eligible patients (*n* = 521) were randomized in a 2:1 ratio to receive the CDK4/6 inhibitor, palbociclib and fulvestrant or placebo plus fulvestrant; all pre- and perimenopausal patients were also given goserelin [43, 51, 52]. In the final analysis, the median PFS was 9.5 months in the palbociclib group vs. 4.6 months in the fulvestrant group (*p* < 0.0001) [43]. Palbociclib-based therapy was well tolerated [53].

The third CDK4/6 inhibitor, abemaciclib, has been tested in premenopausal women in second-line settings. In the global, phase III, randomized, double-blind, placebo-controlled MONARCH-2 study, 699 post- and pre−/perimenopausal women with HR+ HER2 − mBC, who had progressed while receiving endocrine therapy, were randomized to receive in a 2:1 ratio, abemaciclib plus fulvestrant (16% pre−/perimenopausal) or fulvestrant plus placebo (18.8% pre−/perimenopausal). Pre−/perimenopausal women received a GnRH agonist [26] The PFS was noted to be significantly higher in the abemaciclib group (16.9 vs. 9.3 months in fulvestrant group; HR, 0.536; 95% CI, 0.445 to 0.645; *p* < 0.0001) [54].

The mechanism of action of the above three CDK 4/6 inhibitors is directed toward the suppression of RB phosphorylation resulting in a G1 phase arrest, and thus inhibiting cell proliferation [55]. Of the three inhibitors, abemaciclib appears to affect the CDK4 protein, while palbociclib and ribociclib affect both CDK4 and CDK6. Despite similar mechanism of action, the dose limiting toxicities (DLT) differ among these agents, with neutropenia being the DLT for palbociclib, fatigue for abemaciclib, and neutropenia, asymptomatic thrombocytopenia, mucositis, pulmonary embolism, hyponatremia, QTcF, prolongation (> 500 ms), increased creatinine being the DLTs for ribociclib [56]. The most common toxicities of any grade observed in pivotal trials were neutropenia, leukopenia, fatigue and nausea for palbociclib [42, 43], creatinine increase, diarrhea, fatigue, and neutropenia for abemaciclib [24, 54, 57] and neutropenia, nausea, infections, fatigue and diarrhea for ribociclib [28, 35]. All the three inhibitors are given in 4-week cycles with a week-long take off; all are orally administered and undergo metabolism by liver. Palbociclib and ribociclib can be administered once daily, owing to longer half-life, while abemaciclib can be administered twice daily [56].

#### Clinical question 4

Is there a better first-line standard of care than AIs for the treatment of HR + HER2 − mBC?

The evolving treatment landscape of HR + HER2 − mBC has paved the way for the following treatment options other than conventional AIs and tamoxifen in the first-line setting.

### Fulvestrant

In the phase III FALCON trial, fulvestrant single-agent therapy resulted in a significantly longer PFS vs. anastrozole (16.6 vs. 13.8 months, respectively; HR, 0.797; 95% CI, 0.637–0.999; *p* = 0.0486) in postmenopausal women with HR+ HER2− de novo mBC. The PFS benefit with fulvestrant was more evident in patients with nonvisceral disease (22.3 vs. 13.8 months with fulvestrant vs. anastrozole; HR, 0.59; 95% CI, 0.42–0.84) [23] (Table 1), and was consistent in both Asian and non-Asian patient populations [58]. A recent retrospective analysis (*n* = 120) and a meta-analysis (*n* = 3168) also reported similar results, with better efficacy noted for fulvestrant vs. AIs for the treatment of HR + mBC in the first-line setting [59, 60]. In another recent meta-analysis that analyzed the survival benefit of first-line endocrine therapy in visceral vs. nonvisceral HR + mBC, it was noted that fulvestrant had a survival benefit over AIs in patients with nonvisceral disease. Further, the benefits with fulvestrant were noted to be better in HR + mBC patients with nonvisceral vs. visceral disease [61].

For tumors with PIK3CA mutations that have progressed during or after aromatase inhibitors, the addition of PI3K inhibitor, alpelisib, to fulvestrant was associated with improved PFS; mTOR inhibitor everolimus add-on to endocrine therapy has also shown PFS improvements in the endocrine-resistant setting [62].

### Fulvestrant + Anastrozole

The results of the FACT and SWOG studies, comparing the combination of fulvestrant plus anastrozole vs. anastrozole for the treatment of HR + HER2 − mBC in first-line settings, have been conflicting. While FACT revealed no significant TTP benefit with the combination, SWOG revealed significantly superior PFS benefit with the combination (15 months vs. 13.5 months with anastrozole; *p* = 0.007) [24, 25]. The PFS benefit with the combination in the SWOG study was more evident in patients with no prior adjuvant tamoxifen therapy [25]. From the latest final survival results of the SWOG study, the median OS with the combination was noted to be significantly higher than that with anastrozole-alone therapy (49.8 vs. 42 months, respectively; *p* = 0.03). The OS benefit was more prominent in patients who had not received prior tamoxifen (Table 1) [26].

### CDK4/6 inhibitor + endocrine therapy

Another emerging strategy, with better survival benefits over single-agent endocrine therapy for the first-line treatment of HR + HER2 − mBC, is the combination of CDK4/6 inhibitor with endocrine therapy (AI/fulvestrant/tamoxifen). The phase III, randomized, placebo-controlled studies that assessed the combination of CDK4/6 inhibitor plus NSAI vs. NSAI monotherapy are PALOMA-2, MONALEESA-2, MONARCH-3, and MONALEESA-7. While the first three were conducted among postmenopausal women in first-line settings, MONALEESA-7 was conducted among premenopausal women in both first- and second-line settings. The PFS was found to be significantly longer with the combination vs. NSAI monotherapy in all four studies (Table 1) [27, 29–34, 36, 56]. However, this combination may not be a suitable first-line option in patients relapsing within 12 months from the end of adjuvant AI therapy [9].While the subgroup analysis of MONARCH-3 revealed a significantly higher PFS benefit with the combination of abemaciclib plus NSAI in patients with liver metastases, PR-negative tumors, high-grade tumors, or shorter treatment-free interval, the PFS benefit was substantially longer with NSAI monotherapy in patients with a performance status of 0 and patients with bone-only disease [63]. On the contrary, the subgroup analysis of PALOMA-2 revealed a substantial PFS benefit with the combination of palbociclib plus letrozole in patients with low disease burden such as nonmeasurable disease, bone-only disease, or single disease site [64].

The phase III, randomized, double-blind, placebo-controlled MONALEESA-3 study assessed the combination of ribociclib plus fulvestrant vs. fulvestrant monotherapy in patients with HR + HER2 − mBC in both first- and second-line settings [35]. The median PFS was found to be significantly improved in the ribociclib vs. fulvestrant-alone group (33.6 vs. 19.2 months, respectively in first-line settings) (Table 1) [37].

The combination of CDK4/6 inhibitor plus tamoxifen was evaluated in the MONALEESA-7 trial and found to have significantly improved PFS vs. tamoxifen monotherapy (22.1 vs. 11 months, respectively) (Table 1) [40].

In summary, all three endocrine backbones tested in combination with CDK4/6 inhibitors in first-line settings (AI, fulvestrant, and tamoxifen) demonstrated comparable and significant improvement in PFS vs. their respective endocrine single-agent therapies. However, evidence is scant regarding later lines of therapy post CDK4/6 inhibitors. In a retrospective study conducted at a single center in the US, the efficacy of palbociclib and subsequent therapy for HR+, HER2- MBC was investigated [65]. Of 104 patients who experienced progression and underwent subsequent therapy after receiving palbociclib, 12 received exemestane plus everolimus combination therapy. In another single-center retrospective study in the US, treatment after disease progression was investigated in patients who received a CDK4/6 inhibitor as first-line therapy (*n* = 81) or second-line therapy (*n* = 55). Ten patients who received a CDK4/6 inhibitor as first-line therapy received everolimus/exemestane after disease progression, and the median time to treatment failure (TTF) in these patients was 13.2 months [66].

Data on specific patient subgroups who may derive larger clinical benefit with the combination of CDK4/6 inhibitors plus endocrine therapy over endocrine monotherapy are currently based on subgroup analyses, and are inconclusive to guide further clinical treatment decision-making.

### Everolimus + AI/Fulvestrant

In the phase II, open-label, single-arm, multicenter BOLERO-4 trial, 202 patients with HR + HER2 − mBC were treated in the first-line setting with the mTOR inhibitor, everolimus plus letrozole; the median PFS with the combination was found to be 22 months [38]. Further, an exploratory analysis of the phase III, double-blind, randomized, placebo-controlled BOLERO-2 trial that assessed the safety and efficacy of this combination vs. exemestane monotherapy in first-line setting also revealed a significant PFS benefit with the combination vs. AI monotherapy [67] (Table 1). The combination of everolimus and fulvestrant significantly prolonged PFS vs. fulvestrant monotherapy (10.3 vs. 5.1 months, respectively, *p* = 0.02) in patients with HR + HER2 − mBC who developed resistance to AI therapy in the adjuvant setting in the PrE0102 trial [68]. However, considering the limited evidence available in support of everolimus + AI/fulvestrant for the treatment of patients with HR + HER2 − mBC in the first-line setting, caution may be exercised while making clinical decisions on the use of this combination in focus treatment settings.

#### Clinical question 5

What factors influence the choice of single-agent endocrine therapy for the first-line treatment of patients with HR + HER2 − mBC?

Factors that may influence the selection of endocrine therapy for the treatment of HR + HER2 − mBC in the first-line setting include the type and duration of adjuvant endocrine therapy; time elapsed from the end of adjuvant endocrine therapy; disease burden and site; menopausal status of the patient; and efficacy, safety, and quality of life with the treatment [7, 9].

The expert panel reviewed emerging data on the efficacy and safety of CDK4/6 inhibitors for the treatment of HR + HER2 − mBC in the front-line setting, to identify the patient population/s who may not be suited for treatment with these agents and may be better candidates for endocrine monotherapy. Although the incidence of side effects—especially, neutropenia, leukopenia, fatigue, anemia, and thrombocytopenia—was noted to be high in patients treated with CDK4/6 inhibitor therapy [69], these drugs have been noted to be superior to endocrine monotherapy, regardless of patient or tumor characteristics [70]. Hence, the expert panel discussed and agreed that single-agent endocrine therapy may be considered in the first-line setting for the treatment of the following groups of patients with HR + HER2 − mBC: (1) elderly patients; (2) patients with oligometastatic disease; (3) asymptomatic patients; and (4) endocrine-sensitive patients. Further, the panel opined that there is no age cut-off for the use of single-agent endocrine therapy in the first-line setting. Among the single-agent endocrine therapies available for the first-line treatment of HR + HER2 − mBC (fulvestrant, tamoxifen, or AI), the choice of agent was found to be influenced by availability, accessibility, cost, regulatory approval status, patient preference, the endocrine agent used in the adjuvant setting, disease-free interval, tolerability with the endocrine agent used in the adjuvant setting, and de novo mBC status.

Over the years, testing for hormone receptor status has been standard in the breast cancer evaluation; however, other biomarkers are also evolving. The prognostic value of different biomarkers, such as ESR1, CDK4, and MAP3K1 are under investigation. PIK3CA mutations have a strong prognostic value for treatment with α-selective and β-sparing PI3K inhibitors, especially in advance breast cancer [71]. Phase II/III studies of single-agent PARP inhibitors (PARPi) have shown encouraging progression-free survival results in patients with BRCA1/2-mutated breast cancer [72, 73]. Determining mutation status in this breast cancer subgroup could potentially expand treatment options beyond the current standard options.

#### Clinical question 6

What is the preferred treatment plan in mBC patients with oligometastatic disease?

According to European guidelines, the treatment of patients with oligometastatic disease should follow a multidisciplinary approach including locoregional treatment [9]. As there is sparse literature on the subject, the expert panel discussed and agreed upon optimizing the treatment plan in these patients on a case-to–case basis. While a treatment approach involving neoadjuvant therapy, surgery, radiation, and adjuvant therapy may be considered for the treatment of young mBC patients with oligometastatic disease, palliative care may be used for the treatment of elderly patients with low-volume metastatic disease. The FDA pooled analysis of the efficacy and safety of CDK4/6 inhibitors has shown similar efficacy in older women (age 75 or older) compared with their younger women. However, older women were associated with more discontinuations and serious AEs [74]. In a meta-analysis of phase II/III RCTs on ET versus combined strategies, combined ET, especially adding CDK4/6 showed an improvement in PFS as first line treatment in mBC as compared to ET alone [75]. Despite uncertain safety profile, novel agents, such as CDK4/6 inhibitor and mTOR inhibitor, are important in metastatic setting.

#### Clinical question 7

What is the preferred second-line endocrine therapy for the treatment of HR + HER2 − mBC?

**Fig. 1:**
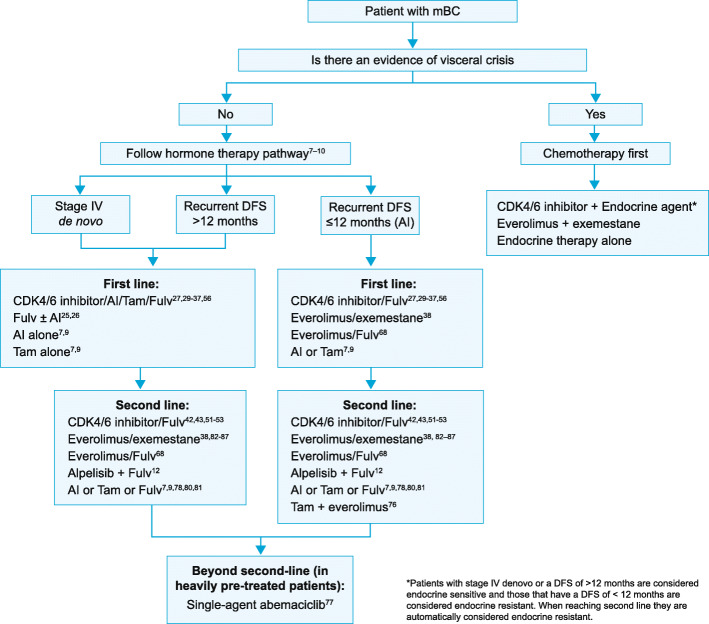
Proposed treatment sequencing for the management of HR + HER2 − mBC

Therapeutic strategy for patients with hormone receptor positive HER2 negative metastatic disease in the second-line setting is based on multiple factors. These include but are not limited to the agents used in the adjuvant and first-line settings; disease-free interval; response to prior hormone therapy; extent of disease; organ function; presence or absence of visceral crisis; endocrine sensitivity; and the presence of a PIK3CA mutation; clinical efficacy and safety of the treatment in the focus setting; patient preference; cost and availability. The use of these factors are in guiding plan of management is illustrated in Fig. 1. Sequential hormonal therapy may be considered only in patients who benefited from prior hormone treatment and have no evidence of immediate life-threatening disease or rapid progression of visceral disease while on adjuvant hormone therapy. Re-initiation of any specific endocrine therapy may be considered only if recurrence with that agent occurred > 12 months from the last treatment. Treatment with CDK4/6 inhibitors should be considered only in those patients without prior exposure to these agents [7, 9].

Endocrine treatment regimens for the management of patients with refractory HR + HER2 − mBC includes single-agent therapy with tamoxifen, steroidal AI, or fulvestrant; or combination therapy with CDK4/6 inhibitors plus fulvestrant, everolimus plus steroidal AI/fulvestrant, or tamoxifen plus everolimus [7, 9, 76]. The combination of alpelisib with fulvestrant may also be considered as a treatment option in this patient population [12]. Single-agent abemaciclib may be the treatment of choice beyond the second line or in heavily pretreated cases [77].

### Single-agent tamoxifen/steroidal AI/Fulvestrant

Single-agent tamoxifen/steroidal AI/fulvestrant has been reported by international guidelines as one of the options for the second-line treatment of HR + HER2 − mBC [7, 9]. Monotherapy with exemestane or fulvestrant may be considered in HR + mBC patients progressing on NSAI therapy [7]. This recommendation is based on the results of the EFECT trial, which revealed comparable outcomes with exemestane vs. fulvestrant in this setting (TTP with both treatments: 3.7 months) [78].Although a Bayesian network meta-analysis comparing fulvestrant 500 mg vs. other therapies for the treatment of HR + mBC following prior endocrine therapy failure reported numerically favorable OS rates with fulvestrant vs. exemestane, additional studies may be needed to derive clinically relevant conclusions [79]. It may be pertinent to mention here that the safety and efficacy of fulvestrant monotherapy in the second-line setting have also been proven in the CONFIRM trial, in which the OS was 26.4 months and PFS 6.5 months with fulvestrant 500-mg regimen (Table 2) [80, 81].

**Table 2:** Summary of key studies for the second-line management of women with HR+, HER2– or unknown HER2 status mBC

First author [year] [reference] [study name]	Treatment arms [n]	Key endpoint outcomes
Chia S et al.*,* 2008 [EFECT]^a,b^ [77]	• Fulvestrant 500 mg IM on day 1, and 250 mg on days 14, and 28, and every 28 days thereafter [total, *n* = 351; second-line setting, *n* = 313] • Exemestane 25 mg OD [total, *n* = 342; second-line setting, *n* = 294]	**Fulvestrant/exemestane** TTP: 3.7 mos in both groups
Di Leo A et al.*,* 2010, 2014 [CONFIRM]^a,b^ [79, 80]	• Fulvestrant 500 mg IM on days 0, 14, and 28, and every 28 days thereafter [total, *n* = 362; second-line setting, *n* = 136] • Fulvestrant 250 mg IM every 28 days [total, *n* = 374; second-line setting, *n* = 177]	**Fulvestrant 500 mg/fulvestrant 250 mg** PFS: 6.5/5.5 mos; *p* = 0.006 OS [final analysis]: 26.4/22.3 mos; *p* = 0.02
Turner NC et al.*,* 2015; Cristofanilli M et al.*,*2016; Turner NC et al. 2018 [PALOMA-3]^c^ [43, 51, 52]	• Fulvestrant 500 mg IM on days 1, 15, and 29 of the first cycle, and every 28 days thereafter + palbociclib 125 mg for 3 wks followed by 1 wk. off [*n* = 347] • Placebo + Fulvestrant [same as Fulvestrant dose in combination] [*n* = 174]	**Fulvestrant + palbociclib/placebo + fulvestrant: Results at final analysis** PFS: 9.5/4.6 mos; *p <* 0.0001 OS: 39.7/29.7 mos; Hazard ratio, 0.72; 95% CI, 0.55–0.94
Sledge GW Jr. et al., 2017; Sledge GW Jr. et al., 2019 [MONARCH-2]^b^ [53, 54]	• Fulvestrant 500 mg IM on days 1 and 15 of the first cycle, and every 28 days thereafter + abemaciclib 200 mg twice-daily, during each 28-day cycle, tapered later to 150 mg [total, *n* = 446; second-line setting, *n* = 171] • Fulvestrant [same as above] + placebo twice-daily [total, *n* = 223; second-line setting, *n* = 85]	**Fulvestrant + abemaciclib/fulvestrant + placebo** PFS: 16.9/9.3 mos; *p <* 0.0001 OS: 46.7/37.3 mos; *p* = 0.013
Slamon DJ et al.*,* 2018 [MONALEESA-3]^b^ [35]	• Ribociclib 600 mg per day on a 3-weeks–on, 1-week–off schedule in 28-day treatment cycles + fulvestrant 500 mg IM on day 1 of each 28-day cycle, with an additional dose on day 15 of cycle 1 [total, *n =* 484; second-line setting, *n* = 236] • Fulvestrant + placebo [total, *n =* 242; second-line setting, *n* = 109]	**Ribociclib + fulvestrant/fulvestrant + placebo** Overall PFS (early relapse + second-line settings): 14.6/9.1 mos; HR 0.571, 95% CI 0.443–0.737 OS (early relapse + second-line settings): 40.3/32.5 mos; HR 0.730, 95% CI 0.530–1.004
Kornblum N et al.*,* 2018 [PrE0102] [total, *n* = 131]^b^ [67]	• Fulvestrant (500 mg on day 1 and day 15 of cycle 1, followed by day 1 of cycles 2 and beyond) + everolimus 10 mg OD • Fulvestrant + Placebo	**Fulvestrant + everolimus/fulvestrant + placebo** PFS: 10.3/5.1 mos; *p =* 0.02 CBR: 63.6%/41.5%; *p =* 0.01
Baselga J et al.*,* 2012, Yardley DA et al.*,* 2013, Piccart M et al.*,* 2014 [BOLERO-2] [81–83]	• Everolimus 10 mg OD + exemestane 25 mg OD [*n* = 485] • Exemestane 25 mg OD [*n* = 239]	**Everolimus + exemestane/exemestane:** PFS [by investigator review]: 7.8/3.2 mos; *p <* 0.0001 OS: 31/26.6 mos; *p =* NS
Jerusalem G et al., 2018 [BOLERO-6] [84]	• Everolimus 10 mg/day + exemestane 25 mg/day [*n* = 104] • Everolimus 10 mg/day [*n* = 103] • Capecitabine 1250 mg/m^2^ twice daily [*n* = 102]	**Everolimus + exemestane/everolimus/capecitabine** PFS: 8.4/ 6.8/ 9.6 mos (everolimus + exemestane vs. everolimus: HR, 0.74; 90% CI, 0.57–0.97) OS: 23.1/ 29.3 mos; Hazard ratio, 1.27; 90% CI, 0.95–1.70
Royce M et al., 2018 [BOLERO-4]^b^ [37]	• Everolimus 10 mg/day + exemestane 25 mg/day [second-line setting, *n* = 50]	PFS in second-line setting: 3.7 mos
Tesch H et al., 2019 [4EVER] [85]	• Everolimus 10 mg/day + exemestane 25 mg/day [total *n* = 299; efficacy evaluation, *n* = 281]	ORR (24 weeks): 8.9% (95% CI, 5.8–12.9%) PFS: 5.6 mos (95% CI, 5.4–6.0 mos)
Cazzaniga ME et al., 2017 [EVA] [total, *n* = 404]^b^ [86]	• Everolimus 10 mg/day + exemestane 25 mg/day	ORR noted in 31.6% of patients Disease control rate noted in 60.7% of patients
Safra T et al., 2018 [87]	• Everolimus 10 mg/day + letrozole 2.5 mg/day [*n* = 72]	PFS: 8.8 mos; 95% CI, 6.6–11.0 mos OS: 22.9 mos; 95% CI, 18.5–28.9 mos
Dickler MN et al., 2017 [MONARCH-1] [76]	• Abemaciclib 200 mg orally every 12 h till disease progression or acceptable toxicity [*n* = 132]	PFS: 6 mos; 95% CI, 4.2–7.5 OS: 17.7 mos; 95% CI, 16.0–Not reached
Andre F et al., 2019 [SOLAR-1] [total, *n* = 572] [12]	• Alpelisib (300 mg/day) + fulvestrant (500 mg every 28 days and once on day 15) • Fulvestrant + placebo	**Alpelisib + fulvestrant vs. fulvestrant + placebo** Investigator-assessed PFS: 11 vs. 5.7 mos; *p* < 0.001 Overall response in patients with *PIK3CA* mutations: 26.6% vs. 12.8%

*HR* Hormone receptor, *HER* Human epidermal growth factor receptor, *mBC* Metastatic breast cancer, *OD* Once-daily, *mos* Months, *NS* Not significant, *PFS* Progression-free survival, *TTP* Time to treatment progression, *OS* Overall survival, *CI* Confidence interval, *IM* Intramuscular, *ORR* Objective response rate, *PIK3CA* Phosphoinositide-3-kinase catalytic alpha-polypeptide, *Wks* Weeks, *EFECT* Evaluation of Faslodex versus Exemestane Clinical Trial, *CONFIRM* Comparison of faslodex in recurrent or metastatic breast cancer, *PALOMA* Palbociclib ongoing trials in the management of breast cancer, *BOLERO* The breast cancer trials of oral everolimus, *MONARCH* The Study of Abemaciclib [LY2835219] Combined With Fulvestrant in Women With Hormone Receptor Positive HER2 Negative Breast Cancer, *MONALEESA* Study of Efficacy and Safety of LEE011 in Postmenopausal Women With Advanced Breast Cancer

^a^HER2 status not reported

^b^Included both first- and second-line settings

^c^Predominantly second-line setting

### CDK4/6 inhibitor + Fulvestrant

The three CDK4/6 inhibitors, palbociclib, abemaciclib, and ribociclib, were evaluated in combination with fulvestrant for the second-line treatment of HR + HER2 − mBC patients in the PALOMA-3, MONARCH-2, and MONALEESA-3 studies, respectively. Significantly superior PFS was noted with the combination vs. fulvestrant monotherapy in all three studies, including in patients with visceral disease [35, 42, 43, 51–53]. (Table 2).

### Everolimus + Fulvestrant/AI

Evidence in support of everolimus plus fulvestrant combination in second-line setting comes from the PrE0102 study, which highlighted a significantly better PFS with the combination vs. fulvestrant monotherapy among 131 postmenopausal women with HR + HER2 − mBC resistant to AI (10.3 vs. 5.1 months, respectively; *p* = 0.01) [68] (Table 2). Everolimus has also been evaluated in combination with exemestane in the second-line setting in several randomized controlled trials and studies in real-world settings. The PFS range in these studies with this combination has been noted to be about 4–8 months [38, 82–86, 88] (Table 2). Further, the safety of this combination was evaluated in the European phase IIIb, expanded-access, multicenter, BALLET study conducted among 2131 heavily pretreated patients with HR + HER2 − mBC (26.4% elderly). The safety profile of the combination in BALLET was found to be consistent with that noted for the combination in BOLERO-2 trial, with no new safety signals [89]. This combination has also been noted to have a favorable impact on bone turnover [87]. The clinical benefit of combining everolimus with the NSAI letrozole was evaluated in a phase II, open-label, single-arm, multicenter trial conducted among 72 postmenopausal women with recurrent HR + HER2 − mBC. The median PFS with the combination was noted to be about 8.8 months, suggesting everolimus plus letrozole to be a plausible option in the focus second-line settings [90] (Table 2).

### Tamoxifen + Everolimus

The tamoxifen plus everolimus (TAMRAD) study revealed a significantly higher CBR, TTP, and OS with everolimus plus tamoxifen combination therapy in second-linesettings vs. tamoxifen alone, but an increased incidenceof side effects [76].

### Alpelisib + Fulvestrant

In the randomized, phase 3, SOLAR-1 trial, the *PIK3CA* inhibitor alpelisib (at a dose of 300 mg/day) was evaluated in combination with fulvestrant vs. fulvestrant monotherapy in patients with HR + HER2 − mBC who had received prior endocrine therapy. The investigator-assessed PFS was significantly longer with alpelisib plus fulvestrant therapy vs. fulvestrant monotherapy (11 vs. 5.7 months, respectively, *p* < 0.001) (Table 2). Further, in patients with *PIK3CA* mutations, the overall response was 26.6% with the combination vs. 12.8% with fulvestrant monotherapy; this benefit was more evident in patients with measurable disease (35.7% vs. 16.2%, respectively) [12].

### Beyond second-line therapy or in heavily pretreated cases: single-agent Abemaciclib

The phase II, single-arm, open-label MONARCH-1 study evaluated the activity and safety of single-agent abemaciclib in 132 women with refractory HR + HER2 − mBC. At the 12-month final analysis, the objective response rate was 19.7%; the clinical benefit rate was 42.4%; the median PFS was 6.0 months, and the median OS was 17.7 months, thus suggesting abemaciclib single-agent therapy as a promising option for heavily pretreated or refractory patients [77] (Table 2).

#### Clinical question 8

Is there an OS benefit with CDK4/6 inhibitors for the treatment of HR + HER2 − mBC?

The use of CDK4/6 inhibitors for the treatment of HR + HER2 − mBC was associated with favorable OS benefit in the PALMOA-3, MONARCH-2, MONALEESA-3, and MONALEESA-7 studies. While fulvestrant was the endocrine partner in the first three studies, the endocrine agent in MONALEESA-7 was NSAI or tamoxifen.

In PALOMA-3, the median OS was found to be longer in the palbociclib plus fulvestrant vs. fulvestrant-alone group, especially in patients with sensitivity to prior endocrine therapy (39.7 versus 29.7 months, respectively; HR, 0.72; 95% CI, 0.55 to 0.94; absolute difference, 10.0 months) [36]. In MONARCH-2, a significant OS benefit of 9.4 months was noted in the abemaciclib group (46.7 vs. 37.3 months in fulvestrant group; HR 0.757, 95% CI 0.606 to 0.945; *p* = 0.0137). The OS benefit was found to be consistent across all subgroups, regardless of menopause status, including in patients with poor prognosis [54]. In MONALEESA-3, the reduction in the relative risk of death with ribociclib was 28% (HR 0.72; 95% CI, 0.57 to 0.92). Overall survival in first-line settings was not reached with ribociclib plus fulvestrant combination vs. 45.1 months with fulvestrant monotherapy (HR 0.700, 95% CI 0.479 to 1.012). In second-line settings, OS was 40.3 months with the combination vs. 32.5 months with fulvestrant monotherapy (HR 0.73, 95% CI, 0.53 to 1.00). The OS benefit with the combination was consistent across all patient subgroups, including in patients with bone-only disease [63].

The OS analysis of MONALEESA-7 revealed a significantly longer OS with ribociclib plus endocrine therapy vs. endocrine therapy alone. The estimated OS at 42 months was 70% in the ribociclib group (95% CI, 63.5 to 76.0) vs. 46.0% in the group receiving endocrine therapy alone, with an estimated 29% lower risk of death (95% CI, 32.0 to 58.9). The overall median OS was not reachable with ribociclib plus endocrine therapy vs. 40.9 months with endocrine therapy alone (HR for death, 0.71; 95% CI, 0.54 to 0.95; *p* = 0.00973). In the subgroup of patients who received NSAI as the endocrine partner, the median OS was not reachable with the combination vs. 40.7 months with endocrine therapy alone (HR, 0.70; 95% CI, 0.50 to 0.98). Further, in the subgroup of patients who received tamoxifen as the endocrine therapy partner, the OS with both combination and endocrine therapy alone was not reachable; at 42 months, the estimated OS was 71.2 and 54.5% in the ribociclib and placebo groups, respectively (HR, 0.79; 95% CI, 0.45 to 1.38) [36].

#### Clinical question 9

In the era of CDK4/6 inhibitors, is there an influence of endocrine resistance on treatment selection in patients with mBC not suitable for single-agent endocrine therapy?

Several mechanisms of endocrine resistance have been implicated in HR + mBC settings, including: (1) mutations in the gene coding for ERα expressed in breast cancer cells, ESR1; (2) amplification of growth receptors, including FGFR1, HER2, EGFR, and IGF1R; (3) activation of phosphoinositide 3-kinase (PI3K)-protein kinase B (AKT)-mTOR pathway; (4) alterations of key cell cycle checkpoints, including hyperphosphorylation of tumor suppressor protein, and amplification/mutation of CDK4; and (5) enhanced levels of basal autophagy. Evidence suggests up to 30% of HR+ mBC may have activating mutations in the ESR1. These may be resistant to AIs but may better respond to ER-targeting therapies, with high dose of tamoxifen/roloxifen or fulvestrant [91, 92].

The use of strategies such as enhanced ER targeting (fulvestrant); increasing target selectivity while decreasing off-target toxicity (CDK4/6 inhibitors); or targeting multiple intracellular pathways and/or multiple points within a pathway (combination therapies) may help overcome endocrine resistance and improve survival rates in the context of HR + mBC [93, 94].

The expert panel discussed and agreed that in the current era of CDK4/6 inhibitors, the upfront use of these drugs or other targeted approaches such as everolimus or alpelisib with well-established survival benefit may help overcome the development of endocrine resistance. While patients with no prior exposure to CDK4/6 inhibitors should be treated upfront with these agents, those who have been treated with prior CDK4/6 inhibitor therapy should be tested for *PIK3CA* mutations. Patients with positive *PIK3CA* mutations should be treated with alpelisib, and those who test negative may be treated with everolimus-based therapy.

#### Clinical question 10

What is the optimal maintenance regimen in mBC patients with visceral crisis treated with chemotherapy and in complete/partial remission?

The expert panel acknowledged that there were no data to guide the management of patients who present in visceral crisis and are treated with chemotherapy and who achieve complete or partial remission. In the absence of data, the panel agreed that combination endocrine therapy with a CDK4/6 inhibitor would be a preferred choice in mBC patients with visceral crisis treated with chemotherapy and in complete/partial remission. In case of no access to CDK4/6 inhibitor, single-agent endocrine therapy may also be a reasonable choice.

#### Clinical question 11

Is there a need for a breast cancer registry?

The expert panel discussed and agreed upon the need or a simple, regional, electronic, prospective breast cancer registry for recording patient demographic and tumor biological characteristics, type of treatment, line of treatment, and details of disease progression, to help understand clinical practice patterns and the commitment of clinicians to optimizing the management of HR + HER2 − mBC in various regions. This regional registry will also help in understanding unmet needs and designing educational activities for clinicians and patients, and in improving accessibility to novel treatments. Existing registries are fragmented and attempt to answer focused questions targeted to specific institutional cohorts within a region. The panel agreed there was a need for a broader registry that would strive to address four main questions:

1. Determine practice patterns across different geographical regions
2. Identify factors that drive decision-making processes, including accessibility variations
3. Compare and contrast PFS and OS across geographical regions
4. Use the derived data to develop strategic policies to aid improved access to care

The panel also agreed that the data proposed to be collected by this broader registry may be aligned in accordance to the updated quality indicators developed by the European Society of Breast Cancer Specialists (EUSOMA) [95] to further enhance the robustness of the registry. Genomic profiling tests are now currently available to identify the most suitable treatment [96]. The phase 2 non-randomized trials– MATCH (NCT02465060) and TAPUR (NCT02693535) are designed to determine the precision-based treatment strategy based on molecular profiling. Metastatic Breast Cancer Project and CancerLinQ are in progress real-world data repositories which facilitate scientific findings as well as the development of novel therapeutic strategies for mBC by extensively sharing clinical, genomic, molecular, and patient-reported data.

### Proposed sequencing strategies for the management of HR+ HER2− MBC

Based on the reviewed evidence, the expert panel proposed a treatment-sequencing algorithm for the management of HR + HER2 − mBC (Fig. 1). The expert panel also reviewed four selected HR+ HER2 − mBC clinical case scenarios and proposed plausible treatment choices for the management of these scenarios (Table 3).

**Table 3:** Proposed treatment choices for the management of HR + HER2 − mBC in selected case scenarios

Sl. No.	Description of the case scenario	Proposed treatment choices	Other preferred first-line choices	Other preferred second-line choices	Other preferred third-line choices
**Case 1**	A 38-year–old woman with stage II breast cancer (ER +/HER2–) treated with surgery, radiation, 4 cycles of cyclophosphamide followed by 12 weeks of paclitaxel, and AI/goserelin, and with 1-year DFS returns with four liver mets and multiple bone mets. Biopsy reveals ER+/HER2–, ki-67: 50%. What would be the choice of treatment in this patient?	First choice: CDK4/6 inhibitor plus fulvestrant Second choice: Everolimus plus fulvestrant Third choice: Fulvestrant alone	–	Participation in a clinical trial or the use of *PIK3CA* inhibitors in patients with confirmed *PIK3CA* mutations.	–
**Case 2**	A 65-year–old woman with stage I ER+/HER2– breast cancer treated with tamoxifen and with a DFS of 12 years returns with 2 bone and 1 lung mets and low-volume disease. What would be the choice of treatment in this patient?	First choice: CDK4/6 inhibitor plus AI or CDK4/6 inhibitor plus fulvestrant in patients intolerant to AI Second choice: AI or fulvestrant single-agent therapy if CDK4/6 inhibitor is not available	–	–	• Exemestane • Everolimus + Exemestane
**Case 3**	A 32-year–old woman presents with stage IV de novo mBC with a 3-cm breast mass (ER/PR+ and HER2–) and bone mets in the hip, T4, and sacrum. What would be the choice of treatment in this patient?	First choice: CDK4/6 inhibitor plus fulvestrant Second choice: Fulvestrant single-agent therapy if CDK4/6 inhibitor is not available	GnRH-A + AI + CDK4/6 inhibitor	If fulvestrant is used in 1st line, GnRH-A ± tamoxifen or AI	GnRH-A + AI, or a clinical trial of exemestane + everolimus
**Case 4**	A 55-year–old woman with recurrent ER+/HER2– breast cancer who was on adjuvant anastrozole and with a DFS of 5 years presents with extensive liver and bone mets, but not in visceral crisis. What would be the choice of treatment in this patient?	First choice: CDK4/6 inhibitor plus fulvestrant Second choice: Everolimus plus fulvestrant, in case of no access to CDK4/6 inhibitor	–	Everolimus + exemestane	Tamoxifen

*HR* Hormone receptor, *HER* Human epidermal growth factor receptor, *ER* Estrogen receptor, *PR* Progesterone receptor, *mBC* Metastatic breast cancer, *DFS* Disease-free survival, *CDK* Cyclin-dependent kinase, *AI* Aromatase inhibitor, *GnRH-A* Gonadotropin-releasing hormone agonist, *PIK3CA* Phosphoinositide-3-kinase, catalytic, alpha-polypeptide

### Implications for clinical practice / conclusion

In this era of evolving therapeutic landscape of HR + HER2 − mBC, careful selection and sequencing of treatment should be done to improve survival rates and safety outcomes. Factors that influence the optimization of treatment selection and sequencing include disease burden and site; prior adjuvant therapy; response to prior endocrine therapy; disease-free interval; patient profile; menopausal status; and clinical efficacy, safety, and quality of life with the treatment in the focus clinical scenario. Besides, regional factors such as availability, accessibility, cost, regulatory approval status, and patient preference may also influence treatment decisions. Establishing a broader registry establishes the real-world treatment patterns, identify the first-line therapy and other data which enables clinical trials and molecular studies.

## Data Availability

Not applicable

## Abbreviations

AI: Aromatase inhibitor
BCL: Basal cell-like
BCCG: Breast Cancer Cooperative Group
BOLERO: The breast cancer trials of oral everolimus
CDK: Cyclin-dependent kinase
CDK4/6: Cyclin-dependent kinase 4/6
CI: Confidence interval
CONFIRM: Comparison of faslodex in recurrent or metastatic breast cancer
DLT: Dose limiting toxicities
DFS: Disease-free survival
ER: Estrogen receptor
EFECT: Evaluation of Faslodex versus Exemestane Clinical Trial
EORTC: European Organisation for the Research and Treatment of Cancer
EUSOMA: European Society of Breast Cancer Specialists
FALCON: Fulvestrant and AnastrozoLe COmpared in hormonal therapy Naïve advanced breast cancer
FACT: Fulvestrant and Anastrozole Combination Therapy
FU: Follow-up
GnRH: Gonadotropin-releasing hormone
HR: Hazards ratio
HR: Hormone receptor
HER: Human epidermal growth factor receptor
HER2: Human epidermal growth factor receptor 2-negative
IM: Intramuscular
mTOR: Mammalian target of rapamycin
mBC: Metastatic breast cancer
TTF: Median time to treatment failure
mos: Months
NSAI: Nonsteroidal aromatase inhibitor
NS: Not significant
OD: Once-daily
ORR: Objective response rate
OS: Overall survival
PALOMA: Palbociclib ongoing trials in the management of breast cancer
PARPi: PARP inhibitors
PIK3CA: Phosphoinositide-3-kinase catalytic alpha-polypeptide
PFS: Progression-free survival
PR: Progesterone receptor
SWOG: SouthWest Oncology Group
TAMRAD: Tamoxifen plus everolimus
TARGET: Tamoxifen or arimidex randomized group efficacy and tolerability study
TTP: Time to treatment progression
